# Plasma exchange as treatment for acute fatty liver disease of pregnancy

**DOI:** 10.1002/ccr3.3845

**Published:** 2021-01-28

**Authors:** Mohammed Aabdi, Yassine Mellagui, Jamal Ouachaou, Essad Ounci, Houssam Bkiyar, Brahim Housni

**Affiliations:** ^1^ Department of Anesthesiology and Intensive Care Unit Faculty of Medicine and Pharmacy of Oujda Mohammed VI University Hospital Mohammed I University Oujda Morocco

**Keywords:** acute fatty liver, plasma exchange, pregnancy

## Abstract

Acute fatty liver disease of pregnancy AFLP is an obstetrical emergency, with severe complications that may include death. Management of AFLP is challenging and include plasma exchange which helps to improve the prognosis for both mother and fetus and delay liver transplantation.

## Graphical abstract

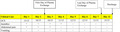

## 1 INTRODUCTION

Acute fatty liver diseases of pregnancy AFLP is a rare life‐threatening situation for both mother and fetus with serious complications. In this paper, we represent a clinical case of a 27 years pregnant woman with AFLP and the management required plasma exchange with clinical and biological improvement.

AFLP is an obstetrical, life‐threatening emergency, with complications for both mother and fetus that might include death.^1^, ^2^, ^3^ The lethality of this disease is due to hepatic and renal impairment.^4^

The clinical and biological manifestations are nonspecific, which makes distinguishing AFLP from other liver diseases of pregnancy such as HELLP syndrome and preeclampsia a challenge for physicians.^1^

In this paper, we report a clinical case of AFLP occurring in the third trimester with hepatic and renal failure with necessary admission to the ICU and 5 sessions of plasma exchange treatment with clinical and biological improvement.

## 2 CASE PRESENTATION

A 27‐year‐old woman, primiparous, with no medical illness, was admitted to the emergency at the 33rd week of pregnancy for progressive asthenia and jaundice.

The physical examination founds a conscious patient, and vital signs: blood pressure 125/80 mm Hg, pulse rate 124 beats/min, respiratory rate 15 cycles/min, and oxygen saturation 96% at room air. The abdominal examination showed no abnormalities.

The complete blood count was as followed: hemoglobin 10 g/dL, thrombocytopenia with a platelet count of 57000/mm^3^, hypoglycemia at 0.55 g/L, hyperbilirubinemia of 82 mg/dL with direct bilirubin of 69 mg/dL; hepatic cytolysis: ASAT and ALAT were 12‐13 times greater than limit, respecti vely, creatinine 41 mg/L, prothrombin ratio 35% and hypoalbuminemia 24 g/l.

**FIGURE 1:**
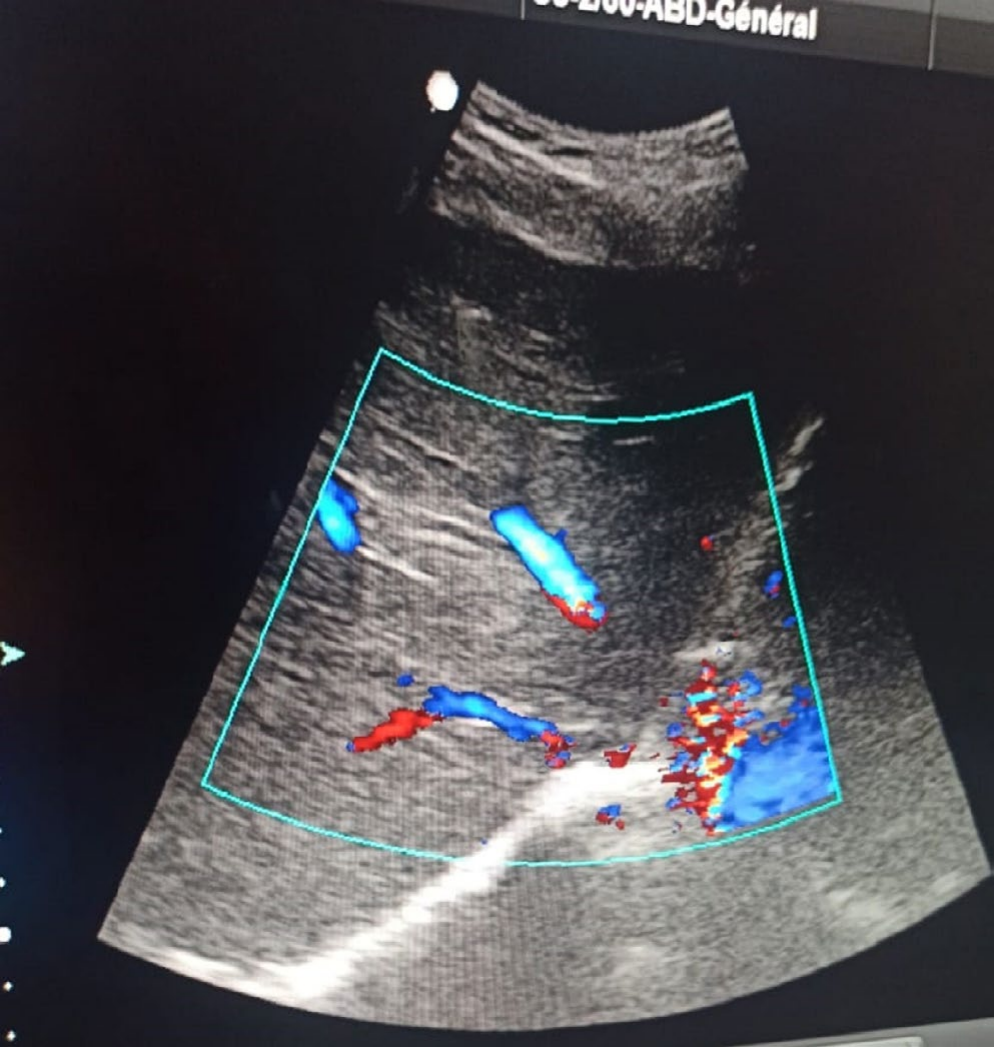
Doppler ultrasound of the liver showing no thrombosis

The liver ultrasound showed no obstruction in the biliary tract or thrombosis in the hepatic vein with normal liver size and no renal dilatation or obstruction (Figure 1).

The obstetrical ultrasonography detected no cardiac activity, so a decision of pregnancy interruption was made. The delivery was normal with no complications.

To rule out the differential diagnosis of hepatic failure, antibodies against Hepatitis A, B, C, and E, and VIG serology were negative.

Acute fatty liver disease of pregnancy was suspected, and the patient was admitted to the ICU where initial management based on fluid resuscitation by glucose 10% solution, and blood product transfusion was initiated. The patient kept worsening both clinically and biologically as follows: neurological deteriorating (GCS 10/15) with normal cerebral CT SCAN, thrombocytopenia of 15 000/mm^3^, so the decision of initiation plasma exchange was made 2 days after ICU admission.

The patient received 5 daily courses of plasma exchange: 2 L were removed and replaced with 1.5 L of plasma each session.

After the fifth session, the patient showed both clinical improvement and biological improvement: she was conscious, and she had platelets 98 000/mm^3^ with normal liver enzymes (Table 1 and 2). She was discharged on day 12.

**TABLE 1:** Evolution of clinical signs

Clinical Case	First day of plasma exchange		Last day of plasma exchange					Discharge
	Day 1	Day 2	Day 3	Day 4	Day 5	Day 6	Day 7	Day 12
GCS	15/15	10/15	10/15	11/15	12/15	12/15	13/15	15/15
Jaundice	+	+	+	+	‐	‐	‐	‐
Abdominal pain	+	+	+	‐	‐	‐	‐	‐
Vomiting	‐	‐	‐	‐	‐	‐	‐	‐

Note

+, Present. ‐, Absent.

**TABLE 2:** Evolution of biological Parameters

Parameters	First day of plasma exchange		Last day of plasma exchange					Discharge
	Day 1	Day 2	Day 3	Day 4	Day 5	Day 6	Day 7	Day 12
Hemoglobin g/dl	10	9,3	9	9	8,5	8,7	9	9,3
Platelet: unit/µm^3^	57 000	15 000	22 000	29 000	53 000	69 000	72 000	98 000
Créatinine mg/l	41	46	37	28	21	16	13	8
Uréa	1.5	2.1	2.0	1.6	1.2	0.7	0.6	0.3
ASAT	300	322	302	210	98	77	70	42
ALAT	325	317	310	215	102	79	69	45
Prothrombin ratio	35%	22%	37%	45%	51%	62%	69%	85%

## 3 DISCUSSION

AFLP is an obstetrical, life‐threatening situation that occurs during the third trimester or immediate postpartum period.^5^ It is a rare disease of pregnancy.^1^ Several risk factors have been identified including multigravida state, male fetus, co‐existing of other liver diseases, previous episodes of AFLP, and underweight mothers.^1^, ^6^, ^7^, ^8^, ^9^, ^10^

The pathophysiology of AFLP is associated with certain genetic impairments in mother and fetus such as long‐chain 3‐hydroxyacyl CoA dehydrogenase (LCHAD) deficiency.^11^ Fetal fatty acid oxidation defects FAOD are associated with a high risk of AFLP. FAOD are caused by a deficiency of enzymes involved in mitochondrial metabolism of fatty acid.^1^ Other fetal fatty acid oxidation defects reported being associated with AFLP such as SCAD (short‐chain acyl CoA dehydrogenase), MCAD (Medium‐chain acyl CoA dehydrogenase), and MTP (mitochondrial trifunctional protein).^11^

Clinical manifestations of AFLP are not specific and are similar to other liver diseases of pregnancy,^12^, ^13^ they include nausea, vomiting, malaise, anorexia, abdominal pain, jaundice, polydipsia, polyuria encephalopathy, liver, and kidney failure.^1^, ^6^, ^13^, ^14^ In our case, the patient presented asthenia, jaundice, liver and kidney failure, and later encephalopathy.

Laboratory tests reveal elevated serum bilirubin level, hypoglycemia, elevated transaminases and ammonia, kidney failure, coagulopathy, and metabolic acidosis.^4^ In our case, the patient had hypoglycemia, cytolysis, kidney, and liver failure.

The differential diagnosis of AFLP is other liver disorders of pregnancy such as HELLP syndrome, and preeclampsia.^15^ The Swansea criteria is a useful tool to help physicians with AFLP diagnosis (Table 3). The diagnosis is positive when 6 of 15 criteria are present in the absence of other diagnoses of liver impairment.^1^

**TABLE 3:** The Swansea criteria

Symptoms:
Vomiting Abdominal pain Polydipsia/polyuria Encephalopathy
Laboratory:
Leukocytosis Hypoglycemia Elevated urate Elevated bilirubin Elevated transaminases Elevated ammonia Coagulopathy Renal impairment
Imaging pathology:
Ascit/bright liver on ultrasound Microvesucular steatosis on liver biopsy

Early delivery remains an important part of the management of this disease, and the liver recovers quickly after ^16^, ^17^; however, considerations should be taken on time and method of delivery ^18^

Immediate induction of labor is associated with a low risk of lethality for both mother and fetus,^19^ and there is no sufficient data for what method of delivery is associated with less risk of bleeding.^20^

Postpartum management is challenging, and special care should be taken to acute liver failure, renal impairment, and disseminated intravascular coagulation ^21^, ^22^

The management requires admission to ICU for monitoring for coagulopathy, massif blood transfusion, and correction of hypoglycemia, mechanical ventilation, dialysis, and plasmapheresis.^2^

Plasma exchange is associated with improved clinical results including maternal mortality.^23^ The mechanism consists of removing endotoxins proteins, intravascular volume, support of coagulation factors, and acid‐base balance, which leads to improvement of hepatic recovery, duration of hospitalization, and decrease in mortality ^24^, ^25^

The indication of plasma exchange in AFLP is reserved for advanced and complicated cases after the failure of other conventional intensive medical management.^4^

Literature review founds that PE was an effective therapy for severe cases of AFLP: 50 of 53 patients recovered in one data, and 37 of 39 improved in another data.^26^, ^27^

## 4 CONCLUSION

AFLP is a life‐threatening situation for both mother and fetus. The diagnosis should be quick for proper management. The Swansea criteria help physicians to distinguish AFLP from other liver diseases of pregnancy. Baby delivery is the main treatment. Plasma exchange is an important tool that can help to improve the prognosis in the severe and complicated case.

## CONFLICT OF INTEREST

The authors do not declare any conflict of interest.

## AUTHOR CONTRIBUTIONS

Dr Aabdi Mohammed: wrote the original draft, conceptualize the data, designed the methodology, involved in software validation and formal analysis, and visualized the data. DR. Mellagui Yassine: wrote and edited the review, involved in formal analysis, visualized the data, approved the version to be submitted. Dr Ouachaou Jamal: wrote and edit the review, involved in formal analysis, visualized the data, and approved the version to be submitted. Dr Es‐Sad Ounci: visualized the data, wrote and edited the review, supervised the data, investigated the data, and approved the version to be submitted. Pr Bkiyar Houssam.: wrote and edited the review, visualized the data, approved the version to be submitted. Pr Housni: administered the project, visualized the data, wrote and edited the review, involved in resources, conceptualized the data, designed the methodology, validated the data, and approved the version to be submitted. All the authors have read and approved the final version of the manuscript.

## PATIENT'S PERSPECTIVE

The patient expressed her satisfaction with the treatment.

## ETHICAL STATEMENT

The study reflects the authors' own research and analysis in a truthful and complete manner.

## Data Availability

The data used to support the findings of this study are available from the corresponding author.
